# QoI Resistance and Phenotypic Variability in *Cercospora beticola* Isolates from Sugar Beet in the Russian Federation

**DOI:** 10.3390/plants15101498

**Published:** 2026-05-14

**Authors:** Vladislav V. Sheremet, Rashit I. Tarakanov, Evgenii S. Mazurin, Anna D. Tokmakova, Svetlana I. Chebanenko, Olga O. Beloshapkina, Peter V. Evseev, Konstantin A. Miroshnikov, Fevzi S.-U. Dzhalilov

**Affiliations:** 1 Department of Plant Protection, Moscow Timiryazev Agricultural Academy, Russian State Agrarian University, Timiryazevskaya Str. 49, Moscow 127434, Russia; svv-7@bk.ru (V.V.S.); svchebanenko@rgau-msha.ru (S.I.C.); beloshapkina@rgau-msha.ru (O.O.B.); dzhalilov@rgau-msha.ru (F.S.-U.D.); 2 Laboratory of Technical Support, Syngenta, Letnikovskaya Str. 2, Moscow 115114, Russia; evgenii.mazurin@syngenta.com; 3 Shemyakin-Ovchinnikov Institute of Bioorganic Chemistry, Russian Academy of Sciences, Miklukho-Maklaya Str. 16/10, Moscow 117997, Russia; anna.zem@mail.ru (A.D.T.); kmi@bk.ru (K.A.M.); 4 Moscow Center for Advanced Studies, Kulakova Str. 20, Moscow 123592, Russia; 5 Laboratory of Molecular Microbiology, Pirogov Russian National Research Medical University, Ostrovityanova 1, Moscow 117997, Russia; petevseev@gmail.com

**Keywords:** sugar beet, *Cercospora beticola*, cercospora leaf spot, azoxystrobin, QoI, *cytB*, G143A, fungicide resistance, phenotypic variability

## Abstract

Sugar beet cercospora leaf spot (CLS), caused by the fungus *Cercospora beticola*, is among the most economically important diseases of sugar beet, and the effectiveness of chemical disease management depends on the susceptibility of pathogen populations to fungicides. In this study, isolates of *C. beticola* from the Russian Federation were characterized based on QoI resistance, molecular detection of the G143A mutation, and phenotypic variability in radial growth, colony morphology, and aggressiveness. A total of 46 leaf samples were surveyed, and 196 isolates were obtained, from which a representative subset of 48 isolates was selected for detailed analysis. The selected isolates were characterized for in vitro radial growth and colony morphology, assessed for aggressiveness on sugar beet leaves, and tested for sensitivity to azoxystrobin based on EC50 values. The G143A mutation was detected by allele-discriminating real-time PCR and confirmed by sequencing of the *cytB* region. The G143A mutation was identified in 41 of 48 isolates (85.4%). In the mycelial bioassay, all isolates had EC50 values above 0.2 µg/mL, and 38 of 48 isolates (79.2%) had EC50 values exceeding 100 µg/mL. Additional validation with SHAM in a subset of 35 isolates did not alter the qualitative interpretation of resistance. Considerable variability was also observed in radial growth rate, aggressiveness, and colony appearance among isolates. These findings indicate widespread QoI resistance in the analyzed Russian isolate collection and provide a structured baseline for regional resistance monitoring and for more cautious use of FRAC 11 fungicides in integrated sugar beet disease-management programs.

## 1. Introduction

Sugar beet (*Beta vulgaris* L.) remains one of the key industrial crops of the temperate climatic zone, and preservation of functional leaf area during the period of sugar accumulation is a critical factor determining yield and raw material quality. The Russian Federation is one of the world’s leading sugar beet-producing countries. According to FAO statistics for 2024, the Russian Federation accounted for approximately 16% of global sugar beet production, highlighting the strategic importance of this crop for national agriculture and the sugar industry [1]. Production is concentrated mainly in several major beet-growing regions, including Krasnodar, Stavropol, Kursk, Lipetsk, Voronezh, and Altai, which were therefore included as key regions in the present study. Because intensive sugar beet production in these regions is associated with regular fungicide use for foliar disease control, they provide a relevant basis for regional monitoring of CLS and QoI resistance. Sugar beet cercospora leaf spot (CLS), caused by *Cercospora beticola* Sacc., is among the most economically important diseases of sugar beet and can lead to substantial losses in both yield and sugar content, especially under daytime temperatures of about 27–32 °C, night temperatures above 15–16 °C, and leaf wetness periods exceeding 10–11 h, which favor repeated cycles of sporulation and secondary infection [2,3,4]. In practical CLS management, integration of agronomic practices, the use of tolerant hybrids, and rational fungicide application play a central role. Under intensive sugar beet cultivation, however, fungicide-based disease management often becomes a decisive component of CLS control and requires maintenance of high efficacy of disease-management programs throughout the season [2,3,5,6]. Long-term and large-scale use of fungicides, especially those with a single-site mode of action, creates pronounced selective pressure on pathogen populations and promotes the accumulation and emergence of resistant genotypes. Among single-site fungicides, quinone outside inhibitors (QoIs; FRAC 11), which target the Qo site of the cytochrome bc1 complex, are particularly prone to resistance development through point mutations in the mitochondrial cytochrome b (*cytB*) gene, including the G143A substitution [7,8]. For *C. beticola*, this has direct practical relevance because QoI fungicides were long regarded as an important component of sugar beet disease-management programs, and in a number of countries reduced field efficacy of QoIs was accompanied by high G143A frequencies in pathogen populations and yield losses [9,10,11]. At the same time, fungicide sensitivity monitoring data emphasize that practical risk assessment should consider not only the presence of a molecular marker such as G143A, but also the phenotypic distribution of sensitivity levels such as EC50, as well as the regional context of fungicide use and sampling structure [12,13].

Accordingly, integration of molecular screening and phenotyping is considered as the most reliable approach for evaluating QoI resistance in applied monitoring programs: the molecular marker reflects the presence of a specific resistance mechanism, whereas phenotypic data record the resulting variability of sensitivity within the population [11,12].

However, for the Russian Federation, systematic data on the phenotypic sensitivity of *C. beticola* to QoI fungicides and on the prevalence of key molecular markers of QoI resistance remain limited, especially regarding the G143A mutation in *cytB*. Under conditions of intensive sugar beet production, the lack of systematic regional resistance data further limits evidence-based adjustment of fungicide programs and may aggravate the already existing risk of ineffective QoI use. Previous studies have shown that phenotypic sensitivity testing and molecular detection of *cytB* mutations are informative components of QoI-resistance monitoring in *C. beticola* [9,12,14,15,16,17,18]. However, comparable regional data for Russian sugar beet-growing areas remain limited. Therefore, monitoring studies integrating EC50-based phenotyping and G143A detection are needed for the major sugar beet-producing regions of the Russian Federation.

The aim of the present study was to characterize *C. beticola* isolates from six major sugar beet-growing regions of the Russian Federation with emphasis on QoI resistance and phenotypic variability. To achieve this goal, the following objectives were addressed: (i) to obtain and identify isolates from sugar beet based on species-level identification; (ii) to characterize their in vitro variability through radial growth and colony morphology; (iii) to assess aggressiveness under controlled leaf inoculation conditions; (iv) to assess the phenotypic sensitivity of isolates to azoxystrobin based on EC50 values; and (v) to screen isolates for the G143A mutation in *cytB* and compare the molecular results with the phenotypic sensitivity data. The results obtained provide a basis for further monitoring of *C. beticola* and for evidence-based management of CLS severity and fungicide resistance in the Russian Federation.

## 2. Results

### 2.1. Sampling Overview and Selection of Representative Isolates

**Figure 1:**
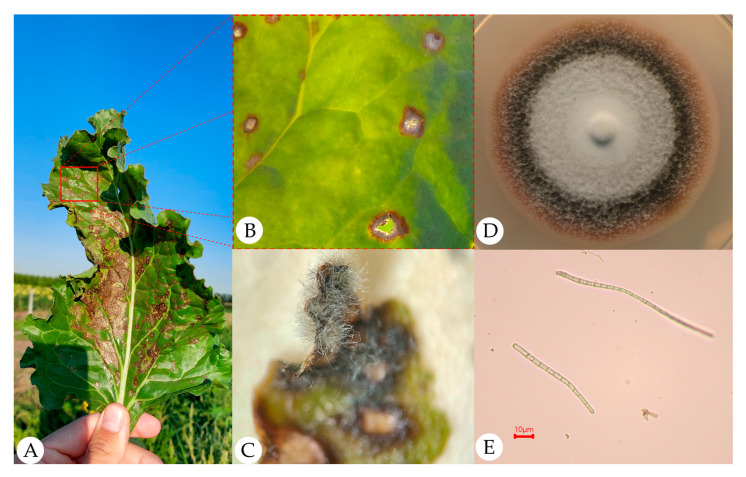
Symptoms of cercospora leaf spot and morphological features of the pathogen *Cercospora beticola* on sugar beet and in pure culture. (**A**) Affected leaf under field conditions in the Krasnodar region; (**B**) typical cercospora leaf spot lesions on the leaf blade; (**C**) sporulation on affected tissue after 5 days of incubation in a moist chamber; (**D**) colony of an isolate on PDA after 10 days of incubation; (**E**) conidia of isolate CLS02-01 (light microscopy, 200×).

During the 2023 growing season, phytosanitary monitoring of sugar beet fields revealed severe damage caused by foliar and stem infections, with characteristic leaf necroses and plant death. Typical round-to-angular necrotic lesions with a lighter center and dark margin were observed on affected leaves, and sporulation developed on the lesion surface. Analysis of symptom appearance and microscopic examination of conidia showed that CLS was the major disease present (Figure 1).

Most of the fields from which leaf samples were collected had been treated with strobilurin fungicides for CLS control. Azoxystrobin (AZO) had been applied to 13 fields, pyraclostrobin (PYR) to 6, trifloxystrobin (TFX) to 5, and kresoxim-methyl (KRE) to 4. In addition, several fields were treated with two strobilurins within one season, namely azoxystrobin + kresoxim-methyl in two fields and azoxystrobin + trifloxystrobin in two fields (Table 1). Overall, during 2019–2023, 46 leaf samples from six regions of the Russian Federation (Altai, Krasnodar, Kursk, Lipetsk, Stavropol, and Voronezh) were examined. From these leaves, 196 monosporic isolates preliminarily assigned to *C. beticola* based on conidial morphology were obtained (Supplementary Table S3). For subsequent in-depth phenotypic and molecular analyses, a representative subset of 48 isolates was selected to cover all sampled regions and available years of isolation (Table 1; Supplementary Table S3), distributed among regions as follows: Altai (*n* = 1), Krasnodar (*n* = 28), Kursk (*n* = 4), Lipetsk (*n* = 2), Stavropol (*n* = 8), and Voronezh (*n* = 5). All isolates were obtained from sugar beet, except CB-WS-3, which was isolated from table beet. One isolate each was obtained in 2019 and 2022, two in 2020, and 44 isolates in 2023.

On PDA, isolates formed slow-growing colonies with dense, velvety–felty aerial mycelium; colony color varied from light gray to dark olive-gray, almost black; and in some isolates, a zone of medium discoloration around the colony (halo) was observed. All isolates formed, from 10–14 days after inoculation on PDA, hyaline, thin-walled, filiform (acicular or slightly curved), elongated, multicellular conidia with numerous transverse septa (3–12 or more), gradually tapering toward the ends, measuring 30–70 × 3–5 µm (Figure 1E); Ct values in the species-specific calmodulin RT-PCR assay [19] were <32.0 (Supplementary Table S1); *rDNA-ITS* sequences showed the highest similarity (>97%) (Supplementary Table S1) to the corresponding sequences of reference *C. beticola* isolates in BLAST searches using the NCBI BLAST web service (https://blast.ncbi.nlm.nih.gov/Blast.cgi, accessed on 4 February 2026), which allowed their assignment to *C. beticola* [3]. Annotated *rDNA-ITS* sequences of *C. beticola* isolates were deposited in the NCBI GenBank database. Characteristics of the isolates and GenBank rDNA accession numbers are given in Supplementary Table S1. Thus, all further phenotypic and molecular analyses were conducted using the 48 isolates described in Table 1.

### 2.2. Phenotypic Variability of Isolates: In Vitro Growth Rate and Colony Morphology

Phenotypic evaluation of the *C. beticola* isolate collection revealed pronounced variability in radial growth rate on PDA. Additional colony appearance traits, including pigmentation and halo expression, were recorded as supplementary descriptors of isolate-level phenotypic variability (Supplementary Table S2; Supplementary Figures S1 and S2).

**Figure 2:**
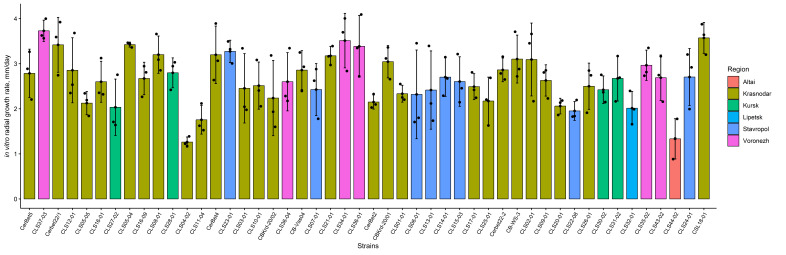
In vitro radial growth rate of *Cercospora beticola* isolates on PDA. Points show individual replicate measurements for each strain, bars indicate mean values, and error bars represent standard deviation. Growth rate is expressed as mm/day. Isolates are arranged in the same order as in Figure 3 to facilitate comparison between in vitro radial growth and aggressiveness, and bar colors correspond to the region of origin. Statistical differences among isolates were assessed by one-way ANOVA followed by Duncan’s multiple range test (α = 0.05); grouping results are provided in Supplementary Table S2.

The radial growth rate of colonies on PDA varied substantially both among individual isolates and among regions of origin (Figure 2). Within the analyzed collection, the trait ranged from 1.33 to 3.15 mm/day, indicating a broad phenotypic range of in vitro radial growth. The highest growth rate was recorded for isolate Cerbet22/1 from Krasnodar and amounted to 3.15 ± 0.61 mm/day, whereas the minimum value was recorded for isolate CLS44-02 from Altai at 1.33 ± 0.45 mm/day. Thus, the collection included both fast-growing and distinctly slow-growing strains, and the difference between the extreme phenotypes was almost threefold. Although in vitro radial growth rate is not interpreted here as a direct proxy of aggressiveness, it was included as an additional phenotypic descriptor to characterize isolate-level variability.

**Figure 3:**
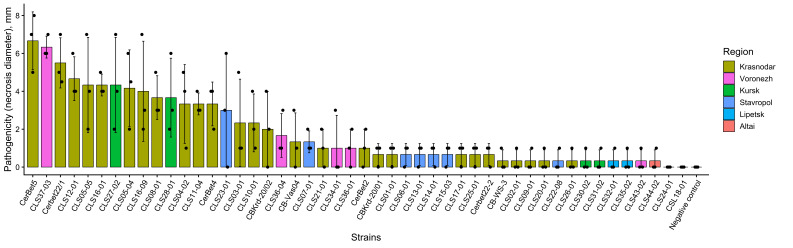
Aggressiveness of *Cercospora beticola* isolates assessed as necrosis diameter on day 7 after inoculation. Bars show mean values for each isolate, points indicate individual replicate measurements, and error bars represent standard deviation. Isolates are arranged in descending order of mean necrosis diameter. The negative control is indicated separately and is shown only as a reference treatment. Statistical differences were assessed among isolates by one-way ANOVA followed by Duncan’s multiple range test (α = 0.05); the negative control was not included as a biological group in the multiple-comparison analysis. Grouping results are provided in Supplementary Table S2.

At the regional level, the distribution of growth rate was also heterogeneous. Median values (min–max) were as follows: Altai (*n* = 1), 1.33 (1.33–1.33); Krasnodar (*n* = 28), 2.70 (1.43–3.15); Kursk (*n* = 4), 2.58 (2.30–2.70); Lipetsk (*n* = 2), 2.61 (2.25–2.97); Stavropol (*n* = 8), 2.63 (2.34–3.00); Voronezh (*n* = 5), 3.08 (2.55–3.12) mm/day. The highest median growth rate was recorded for the Voronezh group; however, the considerable overlap of regional ranges, especially among Krasnodar, Kursk, Lipetsk, and Stavropol, indicates pronounced within-group variability and supports the use of this trait primarily as a descriptive component of the overall phenotypic profile. The estimate for Altai should be interpreted with caution because it is based on only one isolate.

Additional colony morphology traits, including pigmentation and halo expression, were recorded as supplementary descriptors and scored using standardized ordinal scales (Supplementary Table S2; Supplementary Figures S1 and S2). Colony colour varied from light gray to dark olive-gray to nearly black, and halo expression ranged from absent to pronounced. These traits were used as supportive descriptors of isolate-level phenotypic variability and were not treated as standalone primary endpoints. The corresponding isolate-level data for radial growth, colony pigmentation, and halo expression are provided in Supplementary Table S2.

### 2.3. Aggressiveness of Isolates in a Controlled Leaf Inoculation Assay

Aggressiveness was assessed as necrosis diameter on day 7 after inoculation in a controlled leaf inoculation assay and showed pronounced variability within the collection (Figure 3). The negative control was included only to verify the absence of necrosis in the mock treatment and was not treated as a biological group in the multiple-comparison analysis. Necrosis diameter values ranged from 0 to 6.67 mm, including isolates without visually expressed necrotic damage under test conditions and highly aggressive isolates with large necrotic lesions upon inoculation of sugar beet leaves (Supplementary Table S2). Such a broad range indicates substantial heterogeneity in pathogenic potential among the isolates studied.

The maximum aggressiveness was recorded for isolate CerBet5 from Krasnodar (6.67 ± 1.52 mm), corresponding to the upper limit of the overall range. Other highly aggressive isolates included CLS37-03 from Voronezh (6.33 ± 0.57 mm) and Cerbet22/1 from Krasnodar (5.50 ± 1.32 mm), indicating the presence of several strongly expressed aggressive phenotypes with partially overlapping but statistically distinct response classes. The minimum aggressiveness value (0 mm) was recorded for several isolates, including CSL18-01 and CLS24-01 (Figure 3; Supplementary Table S2). The coexistence in one sample of isolates with zero aggressiveness and highly aggressive isolates with necrosis diameters >6 mm further emphasizes the high level of phenotypic heterogeneity of the population in pathogen-related traits. Median aggressiveness values by region (min-max) were Altai (*n* = 1), 0.33 (0.33–0.33); Krasnodar (*n* = 28), 1.67 (0.00–6.67); Kursk (*n* = 4), 2.00 (0.33–4.33); Lipetsk (*n* = 2), 0.33 (0.33–0.33); Stavropol (*n* = 8), 0.67 (0.00–3.00); Voronezh (*n* = 5), 1.00 (0.33–6.33) mm (Figure 3). Notably, extreme values were observed within particular regional groups, for example Krasnodar and Voronezh, whereas the regional ranges overlapped substantially. This indicates that a substantial share of aggressiveness variation is distributed at the level of individual isolates rather than being determined exclusively by geographic origin.

### 2.4. Phenotypic Sensitivity to Azoxystrobin and Molecular Detection of the QoI Resistance Marker G143A in Cercospora beticola

**Figure 4:**
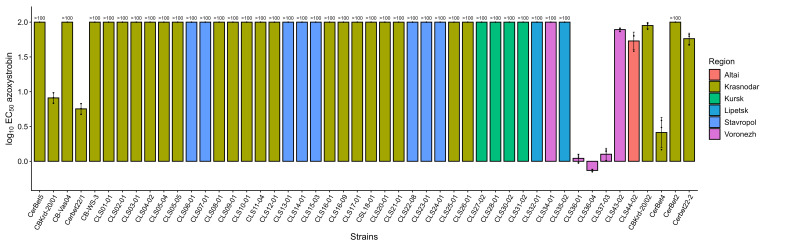
Distribution of sensitivity of *Cercospora beticola* isolates to azoxystrobin according to EC50 values and log10-transformed EC50 values. Bars show mean values, points indicate replicate measurements, and error bars represent standard deviation. Isolates that did not reach 50% growth inhibition at the maximum tested concentration (100 µg/mL) were treated as right-censored observations (EC50 > 100 µg/mL; log10EC50 > 2.00). Based on the EC50 threshold used in the present study (>0.2 µg/mL), all isolates were classified as resistant. The corresponding phenotype for each strain is provided in Table 1.

Sensitivity of *C. beticola* isolates to azoxystrobin was assessed using EC50 values and their log10-transformed values (Figure 4). Because the upper limit of tested concentrations was 100 µg/mL, isolates that did not reach 50% growth inhibition at this concentration were classified as right-censored (EC50 > 100 µg/mL; log10EC50 > 2.00). This approach allowed correct accounting for the extremely low sensitivity of part of the sample within the concentration range used and avoided formally incorrect extrapolation of exact EC50 values beyond the assay range.

In the overall sample, all isolates had EC50 values above 0.2 µg/mL, a threshold previously used in the literature as a benchmark to distinguish sensitive and QoI-resistant isolates. Accordingly, all isolates were classified as resistant to azoxystrobin in Table 1, although substantial quantitative variation in EC50 values was observed among them. At the same time, 38 of 48 isolates (79.2%) had EC50 values exceeding 100 µg/mL, whereas in 10 of 48 isolates (20.8%) the values were measurable within the range of 0.74–89.7 µg/mL. The minimum recorded EC50 value was 0.74 µg/mL for isolate CLS36-04. Because the upper limit of tested concentrations was 100 µg/mL, exact EC50 values could not be determined for 38 isolates which, as a consequence, were treated as right-censored (EC50 > 100 µg/mL).

In an additional validation subset of 35 isolates tested in the presence of SHAM, no sensitive isolates were detected and the qualitative phenotype classification remained unchanged relative to the primary assay (Supplementary Table S4). Although numerical EC50 estimates differed in some isolates, inclusion of SHAM did not alter the overall interpretation of azoxystrobin resistance in the tested subset. Consequently, the data indicate broad prevalence of low sensitivity to azoxystrobin in the collection studied, whereas the internal variability within the group of isolates with EC50 > 100 µg/mL is likely underestimated.

Regional distribution of isolates with EC50 > 100 µg/mL was heterogeneous (Table 2). The highest proportions were observed in Kursk (4/4; 100%), Lipetsk (2/2; 100%), and Stavropol (8/8; 100%), and this phenotype was also highly prevalent in Krasnodar (23/28; 82.1%). In contrast, only 1 of 5 isolates (20%) from Voronezh belonged to this group, whereas the single Altai isolate did not fall into the EC50 > 100 µg/mL category (0/1; 0%). At the same time, these regional proportions should be interpreted descriptively because several groups were represented by only a few isolates, which limits the strength of regional comparisons. Nevertheless, isolates with extremely low sensitivity to azoxystrobin were detected in most sampled regions, indicating broad geographic occurrence of the strongly resistant phenotype in the analyzed isolate collection.

The molecular marker of QoI resistance G143A (allele-discriminating qPCR) was detected in 41 of 48 isolates (85.4%), indicating its wide distribution in the collection studied (Table 1 and Table 2). At the regional level, G143A frequency was as follows: Altai, 1/1 (100%); Krasnodar, 24/28 (85.7%); Kursk, 4/4 (100%); Lipetsk, 2/2 (100%); Stavropol, 8/8 (100%); Voronezh, 2/5 (40%). In most regions, high G143A frequency was combined with a high proportion of isolates with EC50 > 100 µg/mL, which agrees with the expected association between the G143A mutation in *cytB* and QoI resistance. However, these regional frequencies should be interpreted cautiously because several groups were represented by only a few isolates.

At the same time, region-specific and isolate-specific discrepancies between phenotypic and molecular assessment of resistance were detected. The most notable example was the Voronezh sample, in which the frequency of G143A was 40% (2/5), whereas the proportion of isolates with EC50 > 100 µg/mL was only 20% (1/5). For Altai, the single isolate was G143A-positive (1/1; 100%) but did not fall into the EC50 > 100 µg/mL group (0/1), and interpretation of this result is limited by sample size (n = 1). These data show that, at the regional level, phenotype and the G143A marker generally agree but are not in complete correspondence.

Comparison of qPCR results and phenotypic testing at the level of individual isolates confirmed high but not absolute genotype–phenotype concordance. Among G143A-positive isolates, 37 of 41 isolates (90.2%) had EC50 > 100 µg/mL, whereas 4 of 41 isolates (9.8%) retained EC50 < 100 µg/mL values within the range of 55.1–89.7 µg/mL. Among G143A-negative isolates, 6/7 (85.7%) had EC50 < 100 µg/mL; however, one isolate (CerBet2) showed EC50 > 100 µg/mL in the absence of signal for the 143A allele in allele-discriminating qPCR. In total, this corresponded to 43/48 (89.6%) matches and 5/48 (10.4%) mismatches between the two approaches.

The observed mismatches do not diminish the diagnostic value of the G143A marker but indicate that phenotypic expression of QoI resistance in the studied collection is probably not fully explained by G143A status alone, especially when data are aggregated by region and when right-censored EC50 values are present.

**Figure 5:**
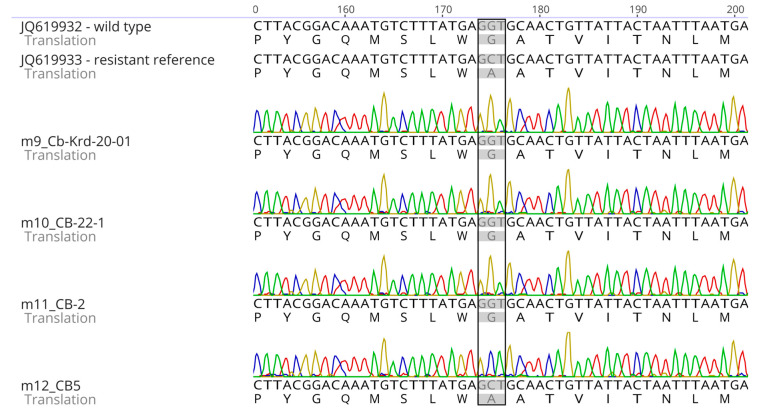
Validation of the G143A substitution in the *cytB* gene of *Cercospora beticola* by sequencing. Reference fragments corresponding to the wild type (JQ619932) and the resistant type (JQ619933), together with representative chromatograms of the analyzed isolates, are shown. The codon corresponding to amino acid position 143 is boxed. Wild-type isolates carry GGT (Gly143), whereas resistant isolates carry GCT (Ala143).

Additional independent verification of the molecular part of the analysis was provided by sequencing of the *cytB* gene fragment containing the G143A mutation site (Figure 5). Diagnostic nucleotide variants corresponding to the wild type and the QoI-associated variant were confirmed in chromatograms. This agrees with the allele-discriminating qPCR results and increases the reliability of G143A marker identification in the sample studied.

Taken together, phenotypic testing of azoxystrobin sensitivity, qPCR detection of G143A, and confirmation of the mutation by sequencing demonstrate the widespread occurrence of QoI resistance in the analyzed *C. beticola* isolate collection (41/48 isolates according to G143A and 38/48 according to the EC50 > 100 µg/mL phenotype), while the degree of correspondence between the molecular marker and phenotypic expression was not absolute. This pattern indicates a high frequency of resistant genotypes while maintaining biologically meaningful phenotypic heterogeneity within the collection.

## 3. Discussion

Sugar beet is a strategically important crop for Russian agriculture and domestic sugar production, and cercospora leaf spot remains one of the major foliar diseases limiting the stability of sugar beet production. Under intensive production systems, the effectiveness of fungicide programs depends not only on correct timing of applications but also on the resistance status of local pathogen populations. Therefore, regional monitoring of *C. beticola* sensitivity to key fungicide groups is essential for evidence-based disease management. The present study provides a combined phenotypic and molecular assessment of QoI resistance in Russian isolates of *C. beticola* and places these resistance data in the context of isolate-level phenotypic variability.

The sampling structure of the study provides a useful baseline for monitoring, although it should not be interpreted as a complete population survey of all Russian sugar beet-growing areas. A total of 46 leaf samples were examined, 196 monosporic isolates were obtained, and a representative subset of 48 isolates was selected for detailed phenotypic and molecular characterization. Species identity was supported by a combination of conidial morphology, species-specific real-time PCR, and ITS sequencing. This multilayer identification workflow reduced the risk of including non-target *Cercospora* taxa and strengthened the reliability of subsequent resistance analyses.

The phenotypic characterization showed that the isolate collection was heterogeneous in radial growth rate, colony morphology, and aggressiveness. Such variability is important because the analyzed collection does not represent a single uniform phenotype, even though QoI resistance was frequent. Radial growth on PDA should not be interpreted as a direct proxy for aggressiveness, and colony appearance should not be treated as a direct indicator of fungicide resistance or pathogenic potential. Instead, these traits provide complementary information on isolate biology and support a broader characterization of the population.

Further comparison of phenotypic traits indicated only partial concordance between in vitro radial growth rate, aggressiveness, and colony morphology. A local tendency toward association of high aggressiveness with halo presence was observed in some isolates, including CerBet5, Cerbet22/1, and CLS12-01. However, this pattern was not universal because pronounced halo formation also occurred in isolates with low or no visible aggressiveness. Likewise, growth rate and aggressiveness showed only partial concordance. These observations indicate that phenotypic variability within the *C. beticola* isolate collection is multicomponent and cannot be reduced to a single trait pattern. Therefore, radial growth, aggressiveness, and colony morphology should be interpreted as complementary descriptors rather than interchangeable indicators of isolate biology.

Aggressiveness also varied substantially among isolates, with necrosis diameter ranging from no visible symptoms to strongly expressed lesions. This variability is relevant for disease-risk interpretation because isolates with similar fungicide-resistance status may differ in their ability to cause visible tissue damage under controlled inoculation conditions. At the same time, the detached leaf assay used here should be regarded as a controlled comparative test rather than a direct substitute for greenhouse or field pathogenicity evaluation. Therefore, aggressiveness data should be interpreted as isolate-level phenotypic descriptors that complement, but do not replace, resistance-monitoring results.

The resistance-related results are strong and internally coherent. The G143A mutation was detected in 41 of 48 isolates, all isolates had EC50 values above 0.2 µg/mL, and 38 of 48 isolates had EC50 values exceeding 100 µg/mL. Taken together, these findings indicate that reduced sensitivity to azoxystrobin is widespread in the analyzed Russian isolate collection. This agrees with previous studies showing that QoI resistance in *C. beticola* is often associated with high frequencies of G143A and reduced efficacy of strobilurin-based control [9,10,11,14,15]. Thus, the present dataset fits the broader international pattern while also providing regionally relevant evidence for the Russian Federation.

An important strength of the study is that QoI resistance was not inferred from a single analytical layer. The molecular marker and the phenotypic assay were broadly concordant, but they were not in complete correspondence. Most G143A-positive isolates belonged to the EC50 > 100 µg/mL group, whereas most G143A-negative isolates had lower measurable EC50 values. At the same time, several mismatches were observed. This is biologically informative, because the marker identifies a specific molecular determinant of resistance, whereas the bioassay records the expressed response of the isolate under the conditions of the test [12,13]. In addition, 38 isolates were right-censored at the upper limit of the assay range, which inevitably reduced finer phenotypic separation within the highly resistant fraction. The most balanced interpretation, therefore, is that G143A is highly informative and strongly associated with resistance but should be interpreted together with phenotypic data rather than used as a complete substitute for them [14,15,16,17,18,20].

The SHAM-based validation supports this conclusion. In the tested subset, no qualitative change in azoxystrobin phenotype classification was observed when results obtained without SHAM were compared with those obtained in its presence. Although numerical EC50 values differed in some cases, the overall interpretation of resistance remained unchanged. This does not remove the limitation that SHAM validation was available only for part of the collection, but it does show that the main resistance signal identified in the study is robust and does not depend on a single assay format [12,14,15]. Additional support for the molecular interpretation was provided by sequencing of the *cytB* region, which confirmed the presence of the G143A substitution in representative isolates. Taken together, these results increase confidence in the conclusion that QoI resistance is genuinely widespread in the analyzed collection.

Several limitations should be considered when interpreting the present results. First, the regional sample was uneven, and several regions were represented by a limited number of isolates; therefore, regional frequencies should be interpreted descriptively rather than as definitive population estimates. Second, SHAM validation was available only for a subset of isolates, although it did not change the qualitative interpretation of resistance. Third, the upper limit of the azoxystrobin assay resulted in a large right-censored fraction, which restricted finer separation among the most resistant isolates. Fourth, QoI resistance was not validated under greenhouse or field fungicide-treatment conditions. This was because the present work was designed as a baseline monitoring study focused on isolate-level phenotyping and molecular detection rather than on evaluation of fungicide efficacy under plant-production conditions. Future studies should combine population-level resistance monitoring with greenhouse and field validation of fungicide performance in order to directly link EC50 values and G143A status with practical disease-control outcomes.

Overall, this study demonstrates that QoI resistance is already widely present in the analyzed *C. beticola* isolate collection from the Russian Federation. For practical sugar beet disease management, this means that FRAC 11 fungicides should not be used as a dominant or repeatedly applied component of CLS control programs in regions where resistant isolates are frequent. Instead, resistance-management programs should rely on continued regional monitoring, rotation of modes of action, effective mixtures, and integration with nonchemical disease-management measures.

## 4. Materials and Methods

### 4.1. Sampling and Obtaining Pure Cultures of Cercospora beticola

**Figure 6:**
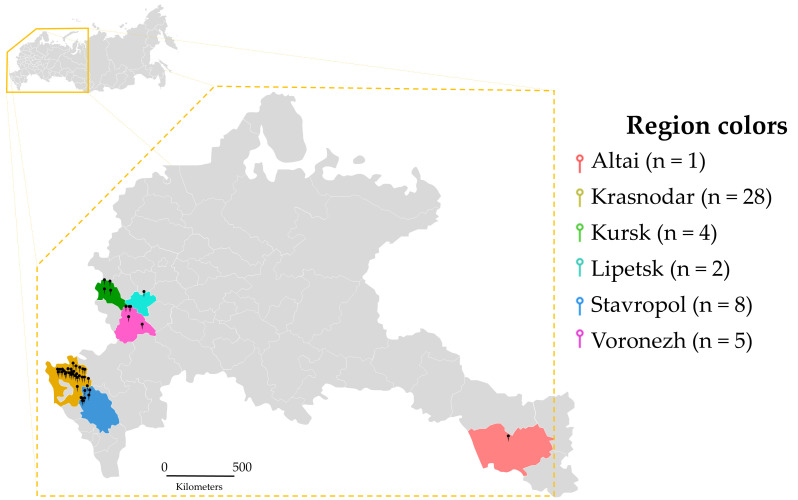
Geographic distribution and regional sampling structure of *Cercospora beticola* isolates included in the study. Sampling regions are indicated by color; the number of isolates in the subset was as follows: Krasnodar-28; Stavropol-8; Voronezh-5; Kursk-4; Lipetsk-2; Altai-1.

Beet leaves with characteristic CLS symptoms were collected under field conditions in the Altai, Krasnodar, Kursk, Lipetsk, Stavropol, and Voronezh regions in 2023. A number of isolates had been collected earlier, in 2019, 2020, and 2022 and were stored in the collection. For each field, a composite sample of 15–20 leaves was formed by selecting leaves from different plants in the central part of the field. Sampling included leaves with a moderate degree of symptoms, approximately 5–10% of the leaf blade area, in the form of round brown lesions, sometimes surrounded by a red-purple ring with sporulating stromatic structures [3], in order to ensure the presence of sporulation while retaining viable tissue. For each sample, the year and region of cultivation, including field coordinates, were recorded, as well as, where available, information on the number of fungicide treatments and active ingredients used. In total, 46 leaf samples were examined, from which 196 monosporic isolates of *C. beticola* were obtained. For in-depth phenotypic and molecular analyses, a representative subset of 48 isolates was selected, including all study regions (Figure 6).

Monosporic isolates were obtained from leaves according to the method of [15] with modifications. To stimulate sporulation, leaves were placed in Petri dishes on sterile moistened filter paper and incubated at 24 °C for 72 h under a 12 h photoperiod with cool white light (CWL; Philips TL-D 58W/865, Philips Lighting, Northants, UK; six lamps per shelf, height 50 cm). After conidial sporulation developed, the spore mass was transferred with a sterile needle into a 1.5 mL tube containing sterile water, vortexed for 15 s, and 50 µL of the suspension was evenly spread over the surface of PDA (HiMedia, Mumbai, India) with a sterile Drigalski spatula. Plates were incubated at 24 °C for 72 h, after which the tip of a single hypha was excised from a developing colony under a stereomicroscope and transferred to fresh PDA medium. To obtain pure cultures, the purification procedure was repeated at least twice. Culture purity was monitored visually based on colony morphology uniformity and the absence of contaminating fungi during successive subcultures. Purified isolates were cultured on PDA for 14 days, then transferred to PDA slants and stored at 4 °C until further analyses. Duplicates of all isolates were also stored at −80 °C in 15% glycerol.

### 4.2. Species Identification of Cercospora beticola Isolates

Species identity of the isolates was established using a combination of species-specific real-time PCR targeting the calmodulin gene, sequencing of the *ITS* region of rDNA, and conidial morphology. Isolates were transferred from slant agar to fresh PDA medium, cultured for 10 days, and then a small fragment of mycelium was taken with a sterile needle from the colony margin and transferred to a sterile tube. DNA was extracted from the collected mycelium using the Phytosorb DNA Extraction Kit (Syntol, Moscow, Russia) according to the manufacturer’s protocol.

For preliminary species-specific screening, amplification of *C. beticola*-specific calmodulin gene sequences was carried out by real-time PCR according to [19]. The reaction mixture contained 10 µL 2.5× Master-mix (2.5× MasEGMIX-2025, Dialat, Moscow, Russia), 0.3 µM of each primer (CbCAL-F and CbCAL-R), 180 nM probe CbCAL-P, 5 ng target DNA, and PCR-grade water to a total volume of 25 µL. Amplification was performed using a CFX96 Real-Time PCR Detection System (Bio-Rad, Hercules, CA, USA). The thermal cycling protocol included initial denaturation at 95 °C for 5 min, followed by 35 cycles of denaturation at 95 °C for 60 s, primer annealing at 52 °C for 30 s, and final extension at 72 °C for 60 s, according to the protocol described by [19]. Fluorescence was measured after primer annealing at each cycle. Threshold values were set manually for the TaqMan^®^ probe. Each isolate was analyzed in three independent replicates. Thus, the identification workflow consisted of an initial species-specific screen by calmodulin real-time PCR, followed by ITS sequencing of positive isolates and confirmation of species-typical conidial morphology.

Isolates showing positive amplification and Ct values < 32 were subjected to in-depth identification by sequencing the internal transcribed spacer (*ITS*) region of the rDNA. For this, reaction mixtures were prepared containing 5 µL 5× Master-mix (5× MasDDTaqMIX-2025, Dialat), 10 pM of each primer (ITS4 and ITS5), 5 ng target DNA, and PCR-grade water to a total volume of 25 µL. Amplification was performed in a T100 thermal cycler (Bio-Rad) according to the program described in [21]. Amplicons were separated by electrophoresis in 1.5% agarose gel, stained with ethidium bromide in 0.5× TBE buffer, and visualized using a Gel Doc XR+ system (Bio-Rad, Hercules, CA, USA). PCR fragments were excised and purified using the ColGen kit (Syntol LLC., Moscow, Russia) according to the manufacturer’s instructions. Sequencing of purified PCR products was performed by the Sanger method using the BigDye Terminator v3.1 Cycle Sequencing Kit (Life Technologies Thermo Fisher, Waltham, MA, USA) and an automated DNA Analyzer 3730 (Thermo Fisher Scientific, Waltham, MA, USA) at Evrogen (Moscow, Russia). The obtained sequences were compared with the GenBank database using the BLASTn algorithm. Conidial morphology, namely the presence on day 10 of culture of hyaline, narrow, straight or slightly curved, multiseptate conidia, was confirmed using a Zeiss Axio Lab.A1 microscope (Carl Zeiss AG, Oberkochen, Germany) at 200× magnification.

An isolate was confirmed as *C. beticola* when the following conditions were met simultaneously: Ct values in the species-specific real-time PCR assay targeting the calmodulin gene [19] were <32.0, the *rDNA-ITS* sequence showed ≥97% similarity to the corresponding sequences of reference *C. beticola* strains in an NCBI BLAST search (accessed 21 January 2026), and the isolate formed conidia typical of the species [3]. Annotated *rDNA-ITS* sequences of *C. beticola* isolates were deposited in the NCBI GenBank database under accession numbers PX944831–PX944878.

### 4.3. Assessment of Radial Growth Rate and Colony Morphology

The growth rate of *C. beticola* isolates was assessed similarly to [22]. For this purpose, a 5 mm mycelial plug was cut with a sterile cork borer from a 10-day-old PDA culture of each isolate, transferred under aseptic conditions in a laminar-flow cabinet with sterile forceps to the center of a new Petri dish containing PDA, sealed with Parafilm, and incubated at 25 ± 0.5 °C. Radial growth of colonies was measured on day 10 in two perpendicular directions using an ADA Mechanic 150 PRO digital caliper (ADA INSTRUMENTS Co LTD., Shenzhen, China). The average radial mycelial growth rate was calculated according to [23] using the following formula:

(1) V = (Dn − D0)/n,

where V is the average growth rate (mm/day); Dn is the colony diameter on day *n* (=10); D0 is the initial diameter (5 mm); and *n* is the number of incubation days.

The experiment was conducted in three replicates (three plates) for each isolate. This trait was used as an additional in vitro phenotypic descriptor and was not interpreted as a direct measure of aggressiveness.

Colony morphology was recorded after 10–14 days of incubation on PDA. Colony pigmentation and halo expression were scored using standardized ordinal scales presented in Supplementary Figures S1 and S2. These traits were used as supplementary descriptors of isolate-level phenotypic variability and were not treated as primary endpoints.

### 4.4. Assessment of Aggressiveness in a Controlled Leaf Inoculation Assay

Aggressiveness of *C. beticola* isolates was evaluated based on symptom development on detached sugar beet leaf discs inoculated with an agar plug containing mycelium according to [24]. Fully expanded, visually healthy sugar beet leaves (hybrid Rekordina KWS, BBCH 33) were collected from fungicide-untreated plants, washed in running tap water with soap for 15 min, surface-sterilized in 0.5% sodium hypochlorite for 30 s, rinsed twice in sterile water to remove sterilant residues, and dried on sterile filter paper to remove excess moisture. Leaf discs 60 mm in diameter were cut from the leaf blades and placed in Petri dishes on moistened sterile filter paper to maintain high humidity. A 5 mm mycelial plug cut from a 10-day-old PDA culture (Bemis Company, Inc., Neenah, WI, USA) was placed in the center of each leaf disc, and the dishes were sealed with Parafilm. Inoculated samples were incubated at 25 ± 0.5 °C in a thermostat, with high humidity maintained by daily moistening of the filter paper with sterile water. Symptoms were recorded 7 days after inoculation, and necrotic lesion size was measured in two perpendicular directions using an ADA Mechanic 150 PRO digital caliper (ADA, Montigny-Lemes, France). Five leaf fragments were inoculated with each isolate, and control leaves received a sterile PDA plug without fungal mycelium. The control treatment was used to verify the absence of necrosis caused by the agar plug or incubation conditions and was not included as a biological group in isolate-level multiple-comparison analysis.

### 4.5. Sensitivity to Azoxystrobin and Detection of the G143A Mutation

#### 4.5.1. Determination of Azoxystrobin EC50

Sensitivity of isolates to azoxystrobin was assessed according to [14,25]. Effective concentrations (EC50) causing 50% inhibition of mycelial growth relative to the control without azoxystrobin were determined. Technical-grade azoxystrobin (strobilurins, QoI; Hubei Kang Bao Tai Fine-Chemical Co., Ltd., Wuhan, China) was used as the active ingredient. Before addition to the medium, azoxystrobin was dissolved in 99.8% dimethyl sulfoxide (DMSO; Komponent-Reaktiv LLC. TD KHIMMED, Moscow, Russia) to prepare stock solutions of 100 mg a.i./mL. Serial dilutions were prepared to obtain the required concentrations. Control plates containing DMSO only were prepared at the same final concentration as in the fungicide treatments, and no inhibitory effect of DMSO on mycelial growth was detected. Prepared solutions were added with automatic pipettes into sterile glass bottles containing PDA medium that had been sterilized in an autoclave and cooled to 47 °C in a water bath, mixed carefully to avoid bubble formation, and poured into sterile Petri dishes (20 mL per plate) using a Levo Plus electric dispenser (DLAB Scientific Co., Ltd., Beijing, China) in a sterile laminar-flow hood. Final azoxystrobin concentrations in PDA were 0 (PDA + DMSO), 0.001, 0.01, 0.1, 1.0, 10.0, and 100 µg/mL. Plates were left for 30 min in the laminar-flow cabinet to solidify. A 5 mm mycelial plug cut from a 10-day-old PDA culture was placed in the center of each plate containing the corresponding azoxystrobin concentration using a sterile needle.

Colony diameter was measured in two perpendicular directions on day 14 of incubation at 25 °C in darkness. Each treatment was established in three replicates. EC50 values were estimated by nonlinear regression in GraphPad Prism 9.2.0 using a four-parameter logistic model with variable slope fitted to the relationship between fungicide concentration and colony diameter. In interpreting the results, a threshold of 0.2 µg/mL, previously used in the literature as a benchmark for distinguishing sensitive and QoI-resistant *C. beticola* isolates [14,26], was applied. The primary mycelial bioassay was performed for the full isolate set (*n* = 48). In addition, a separate validation assay was performed for a subset of 35 isolates in the presence of salicylhydroxamic acid (SHAM) at a final concentration of 60 μg/mL to assess whether inhibition of the alternative oxidase pathway altered the qualitative interpretation of azoxystrobin sensitivity [14]. In the SHAM-tested subset, phenotype classification remained unchanged for all isolates; these data are presented in Supplementary Table S4. EC50 values exceeding the maximum concentration used in the test were recorded as EC50 > 100 µg/mL (right-censored observations) and shown in summary tables and figures as “>100”.

#### 4.5.2. Allele-Discriminating Real-Time PCR for the G143A Mutation in the *cytB* Gene

For preliminary analysis of the presence of the G143A mutation in the cytochrome B (*cytB*) gene, allele-discriminating real-time PCR was performed according to [9]. The reaction mixture contained 10 µL 2.5× Master-mix (2.5× MasEGMIX-2025), 300 nM of each primer and probe (735 (GAGGTCTATACTATGGTTCTTA), 736 (TGTCCTACTCATGGTATTG), 740-SEN (FAM-TGAG[G]TGCAACTGTTATTACTAA-BHQ-1), and 741-RES (HEX-TGAG[C]TGCAACTGTTATTACTAA-BHQ-1)), 5 ng target DNA, and PCR-grade water to a total volume of 25 µL. Probes 740-SEN and 741-RES were synthesized by Evrogen. Amplification was performed on a CFX96 Real-Time PCR Detection System. The thermal profile included initial denaturation at 95 °C for 10 min, followed by 40 cycles of 95 °C for 15 s and 60 °C for 60 s, according to [9]. Fluorescence was measured after primer annealing at each cycle. Threshold values were set manually for each TaqMan^®^ probe. Each isolate was analyzed in three independent replicates. A no-template control (NTC) was included in each run.

#### 4.5.3. Determination of the G143A Mutation in the *cytB* Gene

For precise confirmation of the presence of the G143A mutation in the *cytB* gene, a fragment of the *cytB* gene containing codon 143 was amplified, followed by direct Sanger sequencing of the amplicon according to [9]. Reaction mixtures contained 5 µL 5× Master-mix (5× MasDDTaqMIX-2025), 10 pM of each primer (738 (cytB Forward) and 739 (cytB Reverse)), 5 ng target DNA, and PCR-grade water to a total volume of 25 µL. PCR amplification was carried out in a T100 thermal cycler (Bio-Rad) according to the program described in [9]. Visualization of gene amplification products and Sanger sequencing were performed as described in Section 4.2. Consensus sequence analysis, verification of the presence of the G143A mutation, and its visualization were carried out using Geneious Prime 2026.0.2 (Biomatters Ltd., Auckland, New Zealand). The mutation was recorded when a G→C nucleotide substitution was detected at codon 143, leading to a codon change from GGT (Gly) to GCT (Ala) and the amino acid substitution Gly143Ala [9,10].

### 4.6. Statistical Analysis and Visualization

Statistical processing and visualization were performed in Statistica 12.0 (TIBCO Software Inc., Palo Alto, CA, USA), R (version 4.5.2) in RStudio (version 2026.01.1+403), and GraphPad Prism 9.2.0. For quantitative phenotypic traits (growth rate and aggressiveness), comparisons among isolates were performed using one-way analysis of variance (one-way ANOVA) followed by Duncan’s multiple range test (α = 0.05) to separate mean values; grouping results are presented as letter indices in Supplementary Table S2.

## 5. Conclusions

This study demonstrates the widespread occurrence of QoI resistance in the analyzed isolates of *Cercospora beticola* from the Russian Federation. The G143A mutation was detected in 85.4% of isolates, 79.2% of isolates showed EC50 > 100 µg/mL, and all tested isolates had EC50 values above the resistance benchmark of 0.2 µg/mL. The collection also displayed pronounced phenotypic heterogeneity in radial growth, aggressiveness, pigmentation, and halo expression. These results provide a structured baseline for regional monitoring of QoI resistance in Russia and support more cautious use of FRAC 11 fungicides within integrated sugar beet disease-management programs.

## Figures and Tables

**Table 1:** Origin of *Cercospora beticola* isolates used in the study and indicators of QoI resistance (G143A in *cytB* and sensitivity to azoxystrobin).

Strain Code	Year of Isolation	Sampling Site (Region, District) ^a^	Azoxystrobin EC50, µg/mL ± SD	Azoxystrobin Phenotype ^b^	Ct, (G143), 740-SEN ^c^ ± SD	Ct, (143A), 741-RES ^c^ ± SD
CerBet5	2019	Krasnodar region, Krasnodar district	>100	R	–	24.7 ± 0.7
CBKrd-20/01	2020	Krasnodar region, Krasnodar district	8.23 ± 1.41	R	24.3 ± 0.4	-
CB-Vas04		Krasnodar region, Dinskoy district	>100	R	–	27.8 ± 1.3
Cerbet22/1	2022	Krasnodar region, Krasnodar district	5.73 ± 0.97	R	29.2 ± 0.5	-
CB-WS-3	2023	Krasnodar region, Kanevskoy district	>100	R	–	25.1 ± 1.1
CLS01-01		Krasnodar region, Kanevskoy district	>100	R	–	24.8 ± 0.8
CLS02-01		Krasnodar region, Kanevskoy district	>100	R	–	25.8 ± 1.3
CLS03-01		Krasnodar region, Primorsko-Akhtarsky district	>100	R	–	22.9 ± 0.5
CLS04-02		Krasnodar region, Kanevskoy district	>100	R	–	25.4 ± 0.7
CLS05-04		Krasnodar region, Kanevskoy district	>100	R	–	25.9 ± 1.1
CLS05-05		Krasnodar region, Kanevskoy district	>100	R	–	25.4 ± 0.8
CLS06-01		Stavropol region, Izobilnensky district	>100	R	–	24.9 ± 0.5
CLS07-01		Stavropol region, Novoaleksandrovsky district	>100	R	–	25.3 ± 1.2
CLS08-01		Krasnodar region, Labinsky district	>100	R	–	21.9 ± 0.8
CLS09-01		Krasnodar region, Kurganinsky district	>100	R	–	22.9 ± 0.4
CLS10-01		Krasnodar region, Leningradsky district	>100	R	–	25.2 ± 1.1
CLS11-04		Krasnodar region, Bryuhovetsky district	>100	R	–	25.9 ± 0.3
CLS12-01		Krasnodar region, Leningradsky district	>100	R	–	22.7 ± 0.6
CLS13-01		Stavropol region, Kochubeevsky district	>100	R	–	26.4 ± 0.5
CLS14-01		Stavropol region, Kochubeevsky district	>100	R	–	24.5 ± 0.2
CLS15-03		Stavropol region, Izobilnensky district	>100	R	–	24.9 ± 0.8
CLS16-01		Krasnodar region, Kalininsky district	>100	R	–	22.5 ± 1.4
CLS16-09		Krasnodar region, Kalininsky district	>100	R	–	23.7 ± 0.6
CLS17-01		Krasnodar region, Vyselkovsky district	>100	R	–	23.2 ± 0.6
CSL18-01		Krasnodar region, Novokubansky district	>100	R	–	22.6 ± 0.5
CLS20-01		Krasnodar region, Kurganinsky district	>100	R	–	22.4 ± 0.2
CLS21-01		Krasnodar region, Ust-Labinsky district	>100	R	–	24.2 ± 0.8
CLS22-08		Stavropol region, Novoalexandrovsky district	>100	R	–	22.2 ± 1.4
CLS23-01		Stavropol region, Novoalexandrovsky district	>100	R	–	25 ± 0.6
CLS24-01		Stavropol region, Trunovsky district	>100	R	–	25.9 ± 0.2
CLS25-01		Krasnodar region, Krasnodar district	>100	R	–	23.7 ± 1.2
CLS26-01		Krasnodar region, Krasnodar district	>100	R	–	25.3 ± 0.5
CLS27-02		Kursk region, Fatezhsky district	>100	R	–	24.1 ± 0.1
CLS28-01		Kursk region, Bolshesoldatsky district	>100	R	–	24.0 ± 1.2
CLS30-02		Kursk region, Kursk district	>100	R	–	25.4 ± 0.6
CLS31-02		Kursk region, Zolotuhinsky district	>100	R	–	22.3 ± 1.1
CLS32-01		Lipetsk region, Lebedyansky district	>100	R	–	22.8 ± 1.5
CLS34-01		Voronezh region, Liskinsky district	>100	R	–	20.8 ± 1.5
CLS35-02		Lipetsk region, Lebedyansky district	>100	R	–	22.3 ± 1.6
CLS36-01		Voronezh region, Ramonsky district	1.11 ± 0.15	R	25.5 ± 1.1	–
CLS36-04		Voronezh region, Ramonsky district	0.74 ± 0.03	R	23.7 ± 1.5	–
CLS37-03		Voronezh region, Ramonsky district	1.28 ± 0.24	R	23.6 ± 0.9	–
CLS43-02		Voronezh region, Vorobyovsky district	78.2 ± 4.25	R	–	26.1 ± 1.6
CLS44-02		Altai region, Pavlovsky district	55.1 ± 14.6	R	–	22.3 ± 1.2
CBKrd-20/02		Krasnodar region, Krasnodar district	89.7 ± 9.22	R	–	24.8 ± 1.2
CerBet4		Krasnodar region, Krasnodar district	2.8 ± 1.22	R	25.5 ± 1.1	–
CerBet2		Krasnodar region, Krasnodar district	>100	R	24.9 ± 1.2	–
Cerbet22-2		Krasnodar region, Krasnodar district	58.5 ± 10.18	R	–	25.2 ± 0.5

Note: ^a^-all isolates were obtained from sugar beet except CB-WS-3, which was isolated from table beet. ^b^-R-resistant; S-sensitive; based on the EC50 threshold used in the present study (>0.2 µg/mL), all isolates were classified as resistant; quantitative EC50 values are shown to illustrate variation among resistant isolates. ^c^-740-SEN (FAM) and 741-RES (HEX) are allele-specific TaqMan probes for codon 143 in mitochondrial *cytB*: 740-SEN corresponds to the wild-type G143 allele, whereas 741-RES corresponds to the mutant 143A allele (G143A). Ct-cycle threshold; «–»-no amplification. Comparison of the primary assay with SHAM-based validation data for a subset of isolates is provided in Supplementary Table S4.

**Table 2:** Regional distribution of azoxystrobin sensitivity phenotypes (EC50) and the QoI resistance marker G143A in *Cercospora beticola* isolates.

Region	*N* (Total Isolates)	*N* (G143A+)	G143A+ (%)	*N* (EC50 > 100 µg/mL) *	EC50 > 100 µg/mL (%)
Altai	1	1	100	0	0
Krasnodar	28	24	85.7	23	82.1
Kursk	4	4	100	4	100
Lipetsk	2	2	100	2	100
Stavropol	8	8	100	8	100
Voronezh	5	2	40	1	20
Total	48	41	85.4	38	79.2

Note: *-Isolates not reaching 50% inhibition at 100 µg/mL were classified as EC50 > 100 µg/mL.

## Data Availability

The original contributions presented in this study are included in the article/Supplementary Material. Further inquiries can be directed to the corresponding author.

## Supplementary Materials

The following supporting information can be downloaded at: https://www.mdpi.com/article/10.3390/plants15101498/s1, Table S1: Identification results of *Cercospora beticola* isolates used in the study; Table S2: Phenotypic characteristics of *Cercospora beticola* isolates (in vitro growth rate, aggressiveness, and colony morphology); Table S3: Geographic structure of the sample and results of monitoring for cercospora leaf spot infection in sugar beet fields in 2019–2023; Table S4: Comparison of azoxystrobin sensitivity estimates obtained without and with SHAM in a validation subset of 35 *Cercospora beticola* isolates; Figure S1: Colony colour score scale (1–4) used to phenotype *Cercospora beticola* strains on PDA medium; Figure S2: Halo score scale (0–3) used to phenotype *Cercospora beticola* strains on PDA medium.
