# Supplementary material for: QoI Resistance and Phenotypic Variability in Cercospora beticola Isolates from Sugar Beet in the Russian Federation
Source: Plants (Basel). 2026 May 14;15(10):1498. doi: 10.3390/plants15101498 (PMC13210556; doi:10.3390/plants15101498)
Supplement: Supplementary file 1 [file plants-15-01498-s001.zip › plants-4222292-supplementary.pdf]

*Article*

# QoI Resistance and Phenotypic Variability in *Cercospora beticola* Isolates from Sugar Beet in the Russian Federation

**Vladislav V. Sheremet [1,2], Rashit I. Tarakanov [1,*], Evgenii S. Mazurin [2], Anna D. Tokmakova [3,5], Svetlana I. Chebanenko [1], Olga O. Beloshapkina [1], Peter V. Evseev [4], Konstantin A. Miroshnikov [3], and Fevzi S.-U. Dzhalilov [1]**

[1] Department of Plant Protection, Russian State Agrarian University—Moscow Timiryazev Agricultural Academy, Timiryazevskaya Str. 49, 127434 Moscow, Russia; svv-7@bk.ru (V.V.Sh); svchebanenko@rgau-msha.ru (S.I.C.); beloshapkina@rgau-msha.ru (O.O.B.); dzhalilov@rgau-msha.ru (F.S.-U.D.)
[2] Laboratory of Technical Support, Syngenta, Letnikovskaya Str. 2, 115114 Moscow, Russia; evgenii.mazurin@syngenta.com (E.S.M.)
[3] Shemyakin-Ovchinnikov Institute of Bioorganic Chemistry, Russian Academy of Sciences, Miklukho-Maklaya Str. 16/10, Moscow 117997, Russia; anna.zem@mail.ru (A.D.T.); kmi@bk.ru (K.A.M.)
[4] Laboratory of Molecular Microbiology, Pirogov Russian National Research Medical University, Ostrovityanova 1, 117997 Moscow, Russia; petevseev@gmail.com (P.V.E.)
[5] Moscow Center for Advanced Studies, Kulakova Str. 20, 123592 Moscow, Russia
* Correspondence: r.tarakanov@rgau-msha.ru

## Supplementary files.

**Supplementary Table S1**. Identification results of *Cercospora beticola* isolates used in the study.

| Isolate ID | Leaf sample code | Isolate code | Host cultivar / hybrid | Field fungicide applications (no.: active ingredient) | Genbank rDNA-ITS No. | BLAST Identify to deposited sequence OP390241.1, % | Ct value ± SD in species-specific RT-PCR |
|---|---|---|---|---|---|---|---|
| 1 | N/A[a] | CerBet5 | N/A | | PX944831 | 98.6 | 29.42±1.11 |
| 2 | N/A | CBKrd-20/01 | N/A | | PX944832 | 98.4 | 26.43±1.28 |
| 3 | N/A | CB-Vas04 | N/A | N/A | PX944833 | 98.6 | 31.87±0.95 |
| 4 | N/A | Cerbet22/1 | N/A | | PX944834 | 98.6 | 28.12±1.37 |
| 5 | N/A | CB-WS-3 | N/A (table beet) | | PX944835 | 99.2 | 30.56±0.89 |
| 6 | 23RUCB001 | CLS01-01 | Saxonia KWS | 1: **AZO**[b]; 2: CYP + EPO + PRO + DIF; 3: **KRE** | PX944836 | 98.2 | 29.34±1.14 |
| 7 | 23RUCB002 | CLS02-01 | Saxonia KWS | 1: **AZO**; 2: CYP + EPO + PRO + DIF; 3: **KRE**; 4: PCZ | PX944837 | 98.2 | 26.78±0.71 |
| 8 | 23RUCB003 | CLS03-01 | Baikal | 1: **AZO**; 2: TEB + DIF + PRO + **KRE**; 3: MAN | PX944838 | 98.2 | 30.92±1.48 |
| 9 | 23RUCB004 | CLS04-02 | Dobrava | 1: **AZO** + CYP; 2: MAN | PX944839 | 98.2 | 28.45±1.09 |
| 10 | 23RUCB005 | CLS05-04 | Bartavella | 1: **PYR** + TEB + CYP; 2: PRO + TEB | PX944840 | 98.2 | 27.54±1.23 |
| 11 | 23RUCB005 | CLS05-05 | Bartavella | | PX944841 | 98.2 | 28.90±0.85 |
| 12 | 23RUCB006 | CLS06-01 | Smart Kalledonia KWS | 1: **AZO** + DIF; 2: **TFX** + PTO; 3: CYP + EPO; 4: CYP + PRO | PX944842 | 98.2 | 27.23±1.16 |
| 13 | 23RUCB007 | CLS07-01 | Federica | 1: **AZO** + DIF; 2: CYP + PRO; 3: CYP + PRO | PX944843 | 98.2 | 27.61±1.07 |
| 14 | 23RUCB008 | CLS08-01 | Smart Kalledonia KWS | 1: **AZO** + CYP + MAN; 2: **TFX** + PTO + MAN + TPM; 3: CYP + PRO | PX944844 | 98.2 | 30.04±1.68 |
| 15 | 23RUCB009 | CLS09-01 | Smart Narnia KWS | 1: DIF + **KRE** + EPO; 2: EPO + CYP + MAN; 3: PRO + TEB + CYP | PX944845 | 98.4 | 29.88±0.45 |

| | | | | | | | |
|---|---|---|---|---|---|---|---|
| 16 | 23RUCB010 | CLS10-01 | Recordina KWS | 1: **AZO** + CYP; 2: PRO + CYP; 3: **AZO** + DIF | PX944846 | 97.9 | 26.21±1.10 |
| 17 | 23RUCB011 | CLS11-04 | Smart Kalledonia KWS | 1: **PYR** + EPI; 2: EPI + CYP; 3: PRO + CYP | PX944847 | 98.9 | 26.49±1.22 |
| 18 | 23RUCB012 | CLS12-01 | Smart Narnia KWS | 1: **PYR** + EPI; 2: EPI + CYP; 3: PRO + CYP; 4: PRO + CYP | PX944848 | 99.3 | 29.11±1.06 |
| 19 | 23RUCB013 | CLS13-01 | Smart Kalledonia KWS | 1: DIF + PRO; 2: PRO + CYP; 3: PRO + CYP | PX944849 | 98.4 | 27.09±0.88 |
| 20 | 23RUCB014 | CLS14-01 | Smart Narnia KWS | 1: DIF + PRO; 2: EPO + CYP; 3: SOL + CYP + PRO; 4: PRO + CYP | PX944850 | 98.2 | 31.15±0.65 |
| 21 | 23RUCB015 | CLS15-03 | Recordina KWS | 1: **AZO** + DIF; 2: CYP + PRO; 3: CAR + FLT | PX944851 | 98.2 | 30.33±1.41 |
| 22 | 23RUCB016 | CLS16-01 | Baikal | 1: CYP + PRO | PX944852 | 98.2 | 31.76±1.19 |
| 23 | 23RUCB016 | CLS16-09 | Baikal | | PX944853 | 98.8 | 30.67±0.97 |
| 24 | 23RUCB017 | CLS17-01 | Wapiti | 1: SPX + TEB + TDM | PX944854 | 98.4 | 28.85±1.04 |
| 25 | 23RUCB018 | CSL18-01 | Nero | 1: **TFX** + CYP; 2: SPX + TEB + TDM; 3: PRO + TEB | PX944855 | 98.4 | 29.72±1.26 |
| 26 | 23RUCB020 | CLS20-01 | Bravissima KWS | 1: **TFX** + CYP; 2: SPX + TEB + TDM; 3: PRO + TEB | PX944856 | 98.4 | 26.55±0.79 |
| 27 | 23RUCB021 | CLS21-01 | Eugenia KWS | 1: **TFX** + CYP; 2: CYP + PRO; 3: CYP + PRO | PX944857 | 98.2 | 27.39±1.13 |
| 28 | 23RUCB022 | CLS22-08 | Alando | 1: **AZO** + DIF; 2: EPO + CYP | PX944858 | 98.2 | 31.42±1.59 |
| 29 | 23RUCB023 | CLS23-01 | Yashin | 1: **PYR** + EPO; 2: SOL + CYP + PRO; 3: CYP + PRO | PX944859 | 98.2 | 31.91±0.52 |
| 30 | 23RUCB024 | CLS24-01 | SY Marvin | 1: BEN + **AZO** + PRO; 2: CYP + PRO | PX944860 | 98.2 | 30.08±1.08 |
| 31 | 23RUCB025 | CLS25-01 | Recordina KWS | no treatment | PX944861 | 98.6 | 28.04±1.31 |
| 32 | 23RUCB026 | CLS26-01 | Maksimella KWS | | PX944862 | 98.2 | 26.17±1.01 |
| 33 | 23RUCB027 | CLS27-02 | Recordina KWS | 1: CYP + PRO | PX944863 | 98.4 | 30.25±0.64 |
| 34 | 23RUCB028 | CLS28-01 | Gulliver | 1: DIF + PRO; 2: CYP + EPO | PX944864 | 98.8 | 29.48±0.93 |
| 35 | 23RUCB030 | CLS30-02 | Recordina KWS | 1: DIF + PRO | PX944865 | 98.2 | 27.83±1.02 |
| 36 | 23RUCB031 | CLS31-02 | Recordina KWS | 1: DIF + PRO; 2: CYP + EPO | PX944866 | 98.2 | 31.59±1.05 |
| 37 | 23RUCB032 | CLS32-01 | Smart Narnia KWS | 1: PRO + CYP | PX944867 | 97.9 | 28.66±1.18 |
| 38 | 23RUCB034 | CLS34-01 | Recordina KWS | 1: PYR + EPO | PX944868 | 97.9 | 26.94±0.91 |
| 39 | 23RUCB035 | CLS35-02 | Recordina KWS | 1: **PYR** + EPO; 2: FLU + PTO; 3: PRO + CYP | PX944869 | 98.2 | 30.71±1.56 |
| 40 | 23RUCB036 | CLS36-01 | Recordina KWS | no treatment | PX944870 | 98.2 | 29.02±1.03 |
| 41 | 23RUCB036 | CLS36-04 | Recordina KWS | | PX944871 | 97.8 | 27.14±0.76 |
| 42 | 23RUCB037 | CLS37-03 | Maksimella KWS | | PX944872 | 97.7 | 31.28±1.34 |
| 43 | 23RUCB043 | CLS43-02 | Recordina KWS | 1: **AZO** + CYP | PX944873 | 98.2 | 28.37±1.21 |
| 44 | 23RUCB044 | CLS44-02 | Leopard | 1: **AZO** + EPO | PX944874 | 98.2 | 31.19±0.82 |
| 45 | N/A | CBKrd-20/02 | N/A | N/A | PX944875 | 97.9 | 26.06±1.07 |
| 46 | N/A | CerBet4 | N/A | | PX944876 | 98.4 | 29.95±1.45 |
| 47 | N/A | CerBet2 | N/A | | PX944877 | 98.1 | 30.41±0.98 |
| 48 | N/A | Cerbet22-2 | N/A | | PX944878 | 97.9 | 27.96±1.12 |

Note: [a]- no information on the leaf sample, host cultivar or field treatments. [b]- AZO, azoxystrobin; BEN, benomyl; CAR, carbendazim; CYP, cyproconazole; DIF, difenoconazole; EPI, epiconazole; EPO, epoxiconazole; FLT, flutriafol; FLU, fluopyram; KRE, kresoxim-methyl; MAN, mancozeb; PCZ, prochloraz; PRO, propiconazole; PTO, prothioconazole; PYR, pyraclostrobin; SOL, solatenol; SPX, spiroxamine; TDM, triadimenol; TEB, tebuconazole; TFX, trifloxystrobin; TPM, thiophanate-methyl. Active ingredients indicated in bold belong to the QoI class.

**Supplementary Table S2**. Phenotypic characteristics of *Cercospora beticola* isolates (*in vitro* growth rate, aggressiveness, and colony morphology).

| Isolate ID | Isolate code | Growth rate | | Aggressiveness (necrosis diameter at 7 days) | | Colony colour | Halo around the colony |
|---|---|---|---|---|---|---|---|
| | | Value, mm/day ± SD | Duncan multiple range test (grouping letters) | Value, mm ± SD | Duncan multiple range test (grouping letters) | | |
| 1 | CerBet5 | 2.72±0.54 | abcdefghij | 6.67±1.52 | a | 2 | 1 |
| 2 | CBKrd-20/01 | 3.12±0.36 | abcdefghi | 0.67±0.57 | ij | 1 | 3 |
| 3 | CB-Vas04 | 2.50±0.43 | abcdefghij | 1.33±1.52 | ghij | 2 | 1 |
| 4 | Cerbet22/1 | 3.15±0.61 | abcd | 5.50±1.32 | abc | 2 | 2 |
| 5 | CB-WS-3 | 2.90±0.53 | abcdefgh | 0.33±0.57 | j | 2 | 0 |
| 6 | CLS01-01 | 2.40±0.19 | efghijk | 0.67±0.57 | ij | 2 | 0 |
| 7 | CLS02-01 | 2.72±0.81 | abcdefgh | 0.33±0.57 | j | 2 | 0 |
| 8 | CLS03-01 | 2.60±0.77 | defghijk | 2.33±2.31 | defghij | 1 | 0 |
| 9 | CLS04-02 | 1.43±0.11 | m | 3.33±2.08 | cdefgh | 3 | 0 |
| 10 | CLS05-04 | 2.60±0.06 | abcd | 4.17±2.02 | bcde | 2 | 0 |
| 11 | CLS05-05 | 2.60±0.26 | hijklm | 4.33±2.51 | bcde | 2 | 0 |
| 12 | CLS06-01 | 2.60±0.98 | efghijk | 0.67±0.57 | ij | 2 | 0 |
| 13 | CLS07-01 | 2.34±0.58 | defghijk | 1.33±0.57 | ghij | 2 | 0 |
| 14 | CLS08-01 | 2.77±0.41 | abcdef | 3.67±1.15 | cdefg | 2 | 0 |
| 15 | CLS09-01 | 2.85±0.35 | bcdefghijk | 0.33±0.57 | j | 3 | 0 |
| 16 | CLS10-01 | 2.88±0.52 | cdefghijk | 2.33±1.53 | defghij | 1 | 2 |
| 17 | CLS11-04 | 1.73±0.32 | klm | 3.33±0.57 | cdefgh | 2 | 0 |
| 18 | CLS12-01 | 2.87±0.72 | abcdefghij | 4.67±1.15 | abcd | 2 | 1 |
| 19 | CLS13-01 | 2.63±0.87 | defghijk | 0.67±0.57 | ij | 2 | 2 |
| 20 | CLS14-01 | 2.65±0.43 | bcdefghijk | 0.67±0.57 | ij | 2 | 0 |
| 21 | CLS15-03 | 2.75±0.55 | bcdefghijk | 0.67±0.57 | ij | 1 | 0 |
| 22 | CLS16-01 | 2.65±0.45 | bcdefghijk | 4.33±0.57 | bcde | 1 | 0 |
| 23 | CLS16-09 | 3.02±0.36 | bcdefghijk | 4.00±2.64 | bcdef | 2 | 1 |
| 24 | CLS17-01 | 2.68±0.29 | defghijk | 0.67±0.57 | ij | 2 | 0 |
| 25 | CSL18-01 | 3.02±0.34 | ab | 0.00±0 | j | 2 | 1 |
| 26 | CLS20-01 | 1.90±0.17 | ijklm | 0.33±0.57 | j | 2 | 0 |
| 27 | CLS21-01 | 3.00±0.21 | abcdefg | 1.00±1.00 | hij | 2 | 2 |
| 28 | CLS22-08 | 2.50±0.21 | jklm | 0.33±0.57 | j | 2 | 0 |
| 29 | CLS23-01 | 3.00±0.25 | abcde | 3.00±3.00 | cdefghi | 2 | 0 |
| 30 | CLS24-01 | 2.63±0.63 | bcdefghijk | 0.00±0 | j | 2 | 0 |
| 31 | CLS25-01 | 2.22±0.53 | ghijklm | 0.67±0.57 | ij | 2 | 1 |
| 32 | CLS26-01 | 2.47±0.51 | cdefghijk | 0.33±0.57 | j | 4 | 0 |
| 33 | CLS27-02 | 2.45±0.63 | ijklm | 4.33±2.51 | bcde | 2 | 0 |
| 34 | CLS28-01 | 2.30±0.33 | abcdefghij | 3.67±2.08 | cdefg | 2 | 1 |
| 35 | CLS30-02 | 2.70±0.31 | defghijk | 0.33±0.57 | j | 1 | 1 |

| | | | | | | | |
|---|---|---|---|---|---|---|---|
| 36 | CLS31-02 | 2.70±0.50 | bcdefghijk | 0.33±0.57 | j | 4 | 1 |
| 37 | CLS32-01 | 2.25±0.37 | jklm | 0.33±0.57 | j | 2 | 1 |
| 38 | CLS34-01 | 3.12±0.60 | abc | 1.00±1.73 | hij | 2 | 1 |
| 39 | CLS35-02 | 2.97±0.34 | abcdefghij | 0.33±0.57 | j | 2 | 1 |
| 40 | CLS36-01 | 3.10±0.69 | abcd | 1.00±1.00 | hij | 2 | 0 |
| 41 | CLS36-04 | 2.75±0.65 | bcdefghijk | 1.67±1.15 | fghij | 2 | 0 |
| 42 | CLS37-03 | 3.08±0.24 | a | 6.33±0.57 | ab | 2 | 2 |
| 43 | CLS43-02 | 2.55±0.51 | bcdefghijk | 0.33±0.57 | j | 3 | 0 |
| 44 | CLS44-02 | 1.33±0.45 | lm | 0.33±0.57 | j | 3 | 0 |
| 45 | CBKrd-20/02 | 2.40±0.83 | fghijkl | 2.00±2.00 | efghij | 2 | 1 |
| 46 | CerBet4 | 2.90±0.64 | abcdef | 3.33±1.15 | cdefgh | 1 | 0 |
| 47 | CerBet2 | 2.95±0.16 | hijklm | 1.00±1.00 | hij | 1 | 0 |
| 48 | Cerbet22-2 | 2.35±0.26 | abcdefghij | 0.67±0.57 | ij | 2 | 0 |

Note: Quantitative traits are presented as mean ± SD (n = 3 replicates per isolate), unless otherwise indicated. Categorical traits (colony colour score and halo score) are presented according to the scales shown in Supplementary Figures S1 and S2.

**Supplementary Table S3.** Geographic structure of the sample and results of monitoring for cercospora leaf spot infection in sugar beet fields in 2019–2023.

| Region | Federal district | Number of leaf samples | Number of isolates | Number of isolates in the subset selected for in-depth analysis |
|---|---|---|---|---|
| Krasnodar | Southern | 17 | 108 | 28 |
| Stavropol | Southern | 9 | 54 | 8 |
| Voronezh | Central | 7 | 13 | 5 |
| Kursk | Central | 5 | 13 | 4 |
| Lipetsk | Central | 5 | 3 | 2 |
| Altai | South Siberia | 3 | 5 | 1 |
| **Total** | | 46 | 196 | 48 |

**Supplementary Table S4.** Comparison of azoxystrobin sensitivity estimates obtained without and with SHAM in a validation subset of 35 *Cercospora beticola* isolates.

| Strain code | Region | Azoxystrobin EC50 without SHAM, μg/mL | Azoxystrobin EC50 with SHAM, μg/mL | Azoxystrobin phenotype (without SHAM) | Azoxystrobin phenotype (with SHAM) | Change in azoxystrobin phenotype classification |
|---|---|---|---|---|---|---|
| CerBet5 | Krasnodar | >100 | >100 | R | R | No |
| CBKrd-20/01 | Krasnodar | 8.23 ± 1.41 | 42.1 ± 5.5 | R | R | No |
| CB-WS-3 | Krasnodar | >100 | 4.5 ± 0.6 | R | R | No |
| CLS02-01 | Krasnodar | >100 | >100 | R | R | No |
| CLS03-01 | Krasnodar | >100 | >100 | R | R | No |

| | | | | | | |
|---|---|---|---|---|---|---|
| CLS04-02 | Krasnodar | >100 | >100 | R | R | No |
| CLS05-04 | Krasnodar | >100 | >100 | R | R | No |
| CLS05-05 | Krasnodar | >100 | >100 | R | R | No |
| CLS06-01 | Stavropol | >100 | >100 | R | R | No |
| CLS07-01 | Stavropol | >100 | >100 | R | R | No |
| CLS09-01 | Krasnodar | >100 | >100 | R | R | No |
| CLS10-01 | Krasnodar | >100 | 98.8 ± 0.4 | R | R | No |
| CLS11-04 | Krasnodar | >100 | >100 | R | R | No |
| CLS12-01 | Krasnodar | >100 | >100 | R | R | No |
| CLS14-01 | Stavropol | >100 | >100 | R | R | No |
| CLS16-01 | Krasnodar | >100 | >100 | R | R | No |
| CLS16-09 | Krasnodar | >100 | 83.2 ± 9.3 | R | R | No |
| CLS17-01 | Krasnodar | >100 | >100 | R | R | No |
| CSL18-01 | Krasnodar | >100 | 84.3 ± 8.3 | R | R | No |
| CLS21-01 | Krasnodar | >100 | >100 | R | R | No |
| CLS22-08 | Stavropol | >100 | 7.8 ± 0.5 | R | R | No |
| CLS23-01 | Stavropol | >100 | >100 | R | R | No |
| CLS24-01 | Stavropol | >100 | 99.6 ± 0.4 | R | R | No |
| CLS25-01 | Krasnodar | >100 | 96.5 ± 2.7 | R | R | No |
| CLS26-01 | Krasnodar | >100 | >100 | R | R | No |
| CLS28-01 | Kursk | >100 | 45.8 ± 2.9 | R | R | No |
| CLS31-02 | Kursk | >100 | 53.4 ± 4.0 | R | R | No |
| CLS32-01 | Lipetsk | >100 | 26.9 ± 2.99 | R | R | No |
| CLS34-01 | Voronezh | >100 | >100 | R | R | No |
| CLS35-02 | Lipetsk | >100 | 94.8 ± 6.9 | R | R | No |
| CLS36-04 | Voronezh | 0.74 ± 0.03 | 7.3 ± 0.6 | R | R | No |
| CLS37-03 | Voronezh | 1.28 ± 0.24 | 0.3 ± 0.04 | R | R | No |
| CLS44-02 | Altai | 55.1 ± 14.6 | >100 | R | R | No |
| CerBet4 | Krasnodar | 2.8 ± 1.22 | 1.0 ± 0.09 | R | R | No |
| CerBet2 | Krasnodar | >100 | 82.8 ±9.6 | R | R | No |

Note: SHAM - salicylhydroxamic acid. The table includes a validation subset of 35 isolates tested both in the absence and presence of SHAM. Phenotype was classified according to the EC50 criterion used in the present study. No qualitative change in azoxystrobin phenotype classification was observed in the SHAM-tested subset. Values shown as >100 indicate right-censored observations exceeding the maximum tested concentration.

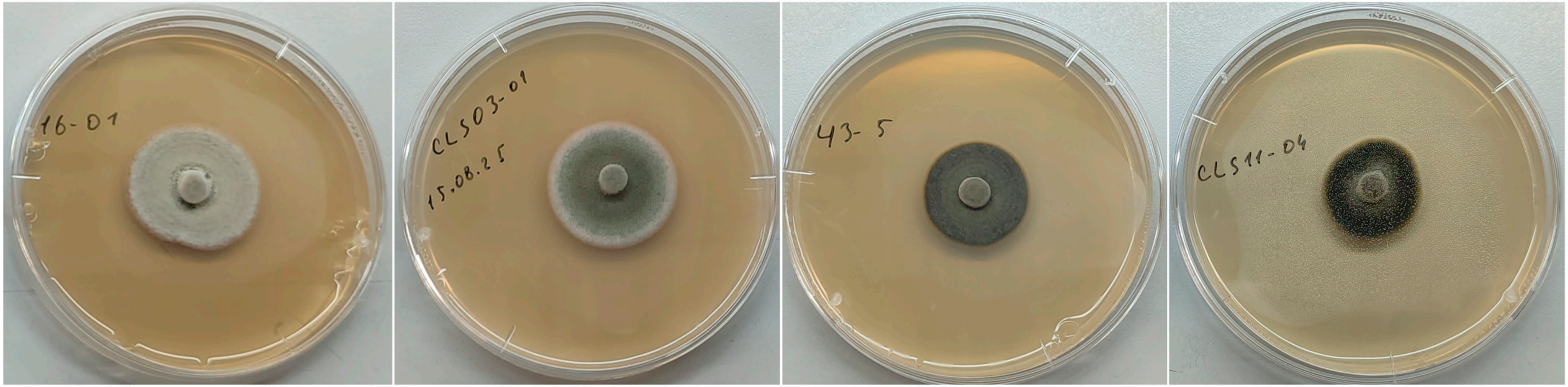


| **Score value** | 1.0 | 2.0 | 3.0 | 4.0 |
| --- | --- | --- | --- | --- |
| **Description** | very light grey-white | grey | dark grey / olive grey | black/black-olive |

**Supplementary Figure S1**. Colony colour score scale (1-4) used to phenotype *Cercospora beticola* strains on PDA medium. Representative colony morphotypes are shown for each ordinal category, where score 1 corresponds to the lightest pigmentation and score 4 to the darkest pigmentation. The colour score was used as a supplementary phenotypic descriptor in the present study.

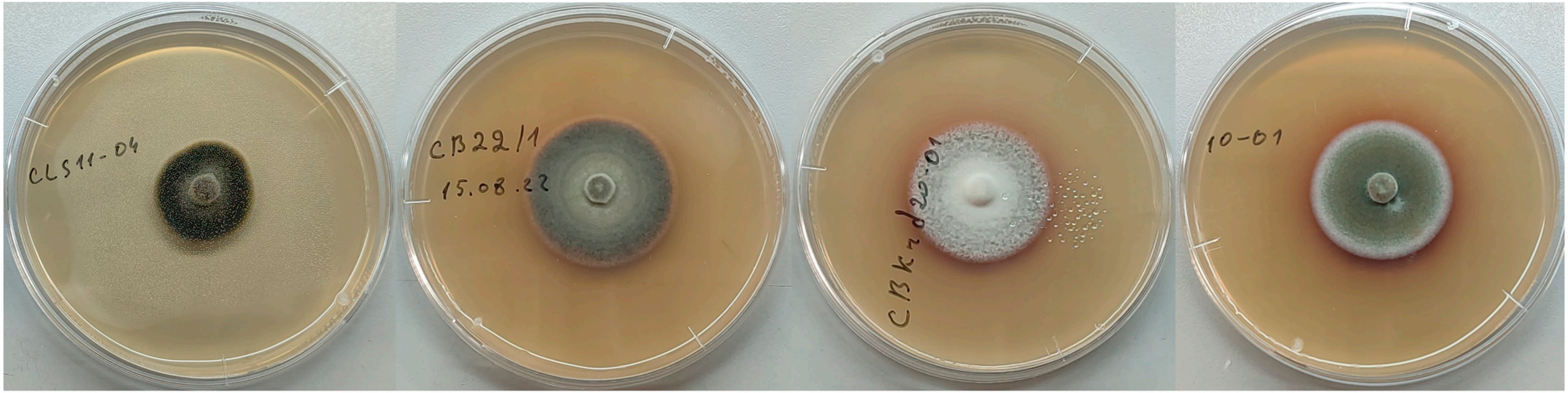


| Score value | 0 | 1.0 | 2.0 | 3.0 |
|---|---|---|---|---|
| Description | there is no halo | faint/thin halo | moderate halo, clearly visible area | wide/strong halo |

**Supplementary Figure S2**. Halo score scale (0–3) used to phenotype *Cercospora beticola* strains on PDA medium. Representative examples are shown for each ordinal category: score 0-no halo; score 1-faint/thin halo; score 2-moderate halo with a clearly visible zone; score 3-wide/strong halo. The halo score was used as a supplementary phenotypic descriptor in the present study.
